# Scraps

**Published:** 1893-09

**Authors:** 


### SCRAPS

PICKED UP BY THE ASSISTANT-EDITOR.

**Sporadic Cretinism.**—Facetious tourist to Swiss peasant: "Is it true that your canton is full of idiots?" Peasant: "Yes, sir—in summer; but they don't stop long."

**Clinical Records** (4).—Doctor (who has been asked to see an ill-developed infant): "What food do you give it?" Mother: "Nawthin'." Doctor: "But it must have some food to live upon. Do you mean to say that you give it nothing?" Mother: "No; it ain't 'ad nawthin'." Doctor: "What! nothing at all?" Mother: "No; nawthin'." Doctor: "Don't you give it any milk?" Mother: "Oh yes; I gives it some milk!" Doctor: "Then why do you say it has nothing?" Mother: "I didn't know what you meant." Doctor: "Do you give it anything besides milk?" Mother: "No; nawthin'." Doctor: "No bread?" Mother: "Yes; I gives it sopped bread." Doctor: "Anything else at all?" Mother: "No; nawthin' else." Doctor: "I suppose when you have your meals it gets something off the table?" Mother: "Oh yes; it picks a bit of meat and wegetables and such like when we gets our dinner."

**For the Coming Session.**—The ten-minute paper has recently become a marked feature in the work of the New York Academy of Medicine. The Section in Pædiatrics was early among the promoters of this innovation, and during the past year has carried out the idea very thoroughly. The result has been a marked increase in the attendance at the meetings, a large number of concise, pithy, and interesting papers, and a wide publication of the proceedings. The instructions to writers of papers formulated by the Chairman, Dr. Northrup, contained a number of apt suggestions, and were somewhat as follows: (1) Hippocrates and Galen may be passed with very slight notice, as they have been for some time dead and their opinions are somewhat obsolete. (2) Scratch out the formal introduction and begin where the subject matter really begins. (3) Condense the body of the paper. (4) End the paper where the subject matter ends, making its action like that of the piston syringe—begin, spatter, stop. As a result of this policy the papers have been unusually practical and to the point.

The genius for sticking to the text is as rare among doctors as among clergymen. It requires courage as well as genius to write a paper upon one subject with a total omission of all one's pet theories upon extraneous matters. Even the most extreme hobby-rider is rarely content with one hobby at a time, but leads along a train of colts of his own breeding, which he is prone to mount at intervals to show off their superior points. A really good hobby-rider is a rarity. Successful papers, almost without exception, are those written with one definite and predominating thought, upon which every fact is brought to bear and toward which every argument is directed. For the ordinary society paper, conclusions alone are, as a rule, sufficient, with pertinent facts so marshalled as to give them proper support. The various minute details of the stages by which these conclusions are reached are usually uninteresting, and had better be touched upon lightly or omitted entirely. It is not alleged that every paper can be made a ten-minute paper, but it is a fact that a large proportion of the papers read at society meetings and published in the journals could be profitably condensed from twenty-five to seventy-five per cent. An expert member of an editorial staff, by remorselessly stripping away the padding, is usually able to make an abstract that will present all the author's ideas and conclusions in one-tenth the space of the original paper. Many a man who has had something of real value to say has first smothered the life out of it with padding and then dug a grave for it and buried it in the midst of a five-column paper compiled from some text-book. It would be far better for medical literature if every man would content himself with writing what he really knew instead of writing what he did not know. One new fact discovered, one new, live, practical idea, is a sufficient subject for one paper, though it may be a short one. Two or three subjects for a single paper will render it weak or actually inert. A shot-gun is adapted to small game, but large game is only brought down with a rifle. A single paper upon a live subject, if it hits the mark squarely, will do more to establish a man's reputation than ten diluted and watery compilations.—*New York Medical Journal.*

**Medical Philology** (VII.)—In the *Promptorium* are the following words:

BLERE YED. *Lippus.*

BLERYDNESSE. *Lippitudo.*

In the 1499 edition the first of the words was printed "blere iyed," and the second "blere iyednesse."

The *Catholicon* has these forms:

to Blere
to be Blerid; *lippire*, *lippescere.*
Blere eede; *lippus.*
a Bleredness; *leppitudo; apifora.*

The British Museum MS. of the *Catholicon* gives *lippire*, *lippiscere*, as the equivalents of "to blere," and had the reading of "Blered" for "Blere eede."

The notes by Mr. Way and Mr. Herrtage are these:

"*Lippus dicitur qui habet oculos lachrymantes cum palpebris euersatis*, blered of the eye." ORT. VOC. In Piers Ploughman the verb to blere occurs, used metaphorically: "He blessede hem with his bulles, and blerede hure eye." "To bleare ones eye, begyle him, *enguigner.*" PALSG.

'*Lippio*, to be pore-blind, sande-blind, or dimme of sight. *Lippitudo*, bleredness of the eyes. *Lippus*, bleare-eyed: hauing dropping eies.' Cooper. '*Lippitudo.* Blerynes of the eye. *Lippio.* To wateryn with the eye.' Medulla. In the Poem of Richard the Redeles (E.E. Text Soc., ed. Skeat), ii. 164, we have *blernyed*=blear-eyed. To blere one's eye is a common expression in early English for to deceive one; thus Palsgrave gives 'I *bleare*, I begyle by dissimulacyon;' and the Manip. Vocab. has 'to blirre, *fallere.*' For instances of this use of the word see Wright's Sevyn Sages, pp. 48, 77, and 100; the Romaunt of the Rose, l. 3912, &c.; Ly Beaus Disconus (in Weber's Met. Rom., vol. ii) l. 1432; Wrights Political Poems, ii. 172; Sir Ferumbras, ed. Herrtage, l. 391, &c.

'For all ower besynes, *bleryd is ower eye.*' *Digby Myst.* p. 92, l. 985.

In *Genesis* xxix. 17, the A.V. reads "Leah was tender eyed; but Rachel was beautiful and well-favoured." Wyclif (1382) has for the first part of the verse, "Lya was with blerid eyen." In *Leviticus* xxi. 20, where Wyclif reads "bleereyed," Coverdale (1535) has "eny blemysh in the eye." The A.V. gives "blemish," and many translators read "a white speck." In *Piers Plowman* (1393) occurs this passage:

"For smoke and smolder
Smyteth in hise eighen
Till he be bler-eighed or blynd."—ed. Wright, 1856, p. 367.

It would seem from the use of the term by Wyclif and Langland that its earlier reference was to dimness of sight, and the theory of its connection with "blurred" would seem a reasonable one. Then came later its application to the condition of things described in the *Ortus* and for which *lippitudo* found in modern text-books is now used. To-day the term "blear-eyed" usually denotes this raw condition of the eyelids, and is so described in many dictionaries. But "dim-sighted" is the only definition given by Professor Skeat.

With a change of literal signification, the early meaning would naturally live on in metaphorical language, and of this many instances are to be found in English literature of much later date than those given by Mr. Herrtage. Shakspere uses the term in its threefold application—its figurative meaning, its reference to dimness of sight, and its sense of disfigurement of countenance. In *The Taming of the Shrew* (v. 1. 120), Lucentio, having Bianca as his wife and alluding to the deceptions practised on her father, says to him, "Counterfeit supposes blear'd thine eyne." Brutus, referring to Coriolanus (II. 1. 221-2), says "All tongues speak of him, and the bleared sights Are spectacled to see him." When Bassanio is about to make choice of the caskets, Portia likens Nerissa and the attendants to "the Dardanian wives, With bleared visages," where the allusion is to eyes made red with weeping.

